# Information-Sharing Behavior on WeChat Moments: The Role of Anonymity, Familiarity, and Intrinsic Motivation

**DOI:** 10.3389/fpsyg.2019.02540

**Published:** 2019-11-14

**Authors:** Xi Chen, MingXue Sun, Dong Wu, Xiao Yu Song

**Affiliations:** ^1^School of Business and Tourism Management, Yunnan University, Kunming, China; ^2^Management College, Ocean University of China, Qingdao, China; ^3^School of Economics and Management, Beijing Normal University, Beijing, China

**Keywords:** social networking sites, information sharing, perceived anonymity, intrinsic motivation, familiarity

## Abstract

Information-sharing behavior is affected by identity recognition perception. The current study aims to delve into the impact of familiarity and anonymity on information-sharing behavior, and the mediating role of intrinsic motivations on WeChat Moments. We hypothesized a mediator role of intrinsic motivations in the relationship between an individual’s perceptions and information sharing. Based on the self-determination theory, a model was created and tested using a sample of 531 frequent users. In this study, these users were asked to use WeChat Moments, the most popular mobile private social networking site in China. The results demonstrate the significance of familiarity and identifiability in an interpersonal relationship, when using social networking sites. Moreover, the influence of perceived anonymity on information-sharing behavior, which is entirely mediated by intrinsic motivation has been validated from an empirical perspective. Our findings extend previous studies by showing the totally mediated effect of perceived anonymity on information-sharing behavior on WeChat Moments and the influential mechanism of intrinsic motivation. The results will inform researchers about the importance of incorporating the interpersonal structural features and intrinsic motivation of social networking sites into future studies on online information-sharing behavior. Important ways to promote attention and share information involve building a familiar relationship with communities and equipping oneself with off-line relations. Final indications for future developments are provided, with a special emphasis on the development of these findings in various social networking sites contexts.

## Introduction

With social networking sites becoming more and more critical, individuals all over the world have integrated these sites into their daily practices (Lee and Ma, 2012; Cheung et al., 2015). Social networking sites, abbreviated SNSs, mainly has three functions: To improve the innovation ability of individuals and groups, to explore social behavior change, and to cultivate collective intelligence (Cachia et al., 2007), improving people’s lives, users with low self-esteem and life satisfaction benefit most from the use of SNS (Błachnio et al., 2016), which is positively related to friendship quality (Wang et al., 2014), and personal characteristics, attitudes, motivations, privacy concerns, and self-efficacy may predict the use of SNSs (Wang D. et al., 2015; Wang J.L. et al., 2015; Dienlin and Metzger, 2016; Chua and Chua, 2017).

A long line studies have explored factors that may influence one’s information disclosure on SNSs (Krasnova et al., 2012; Cheung et al., 2015; Shibchurn and Yan, 2015; Zhang and Ling, 2015; Chen et al., 2016; Dienlin and Metzger, 2016; Levontin and Yom-Tov, 2017; Lin and Utz, 2017). On different SNSs, users use different identity construction strategies to find a balance between the exposure and concealment of their real identity (Cunningham, 2013; Chen et al., 2016). Indeed, 81% of Internet users used strategies to protect their real identity, and the average anonymity seeker used between 3 and 4 of these strategies each time (Rainie et al., 2013). The more open the information sharing, the more a social networking site contributes to participatory social capital (Park et al., 2012). However, there is a gap in the research relating to the influence of the concealment of users’ identities on self-disclosure or information-sharing behavior on different SNSs.

According to the different boundaries associated with information presentation, social networking sites can be classified as public and private. According to global social media research released in 2016, Facebook was an open social media platform and the world’s largest SNS. WhatsApp was the world’s second largest SNS and the world’s largest private SNS, which is a kind of acquaintance social networking site, only friends of the user can see his/her moments (Lin et al., 2017). WeChat was ranked as the fifth largest SNS in the world and the second largest private SNS in the world (Chaffey, 2016), becoming one of the world’s most popular social media platforms (Montag et al., 2018). In China, WeChat is the most widely used social networking site, more than 90% of mobile phones have installed WeChat (Wang, 2017), and by the end of 2019, WeChat had over 1 billion active users around the world.

Internet users around the world spend an average of 2 h and 22 min a day on social networking sites, and have an average of 8.5 social media accounts and tend to use each platform for different purposes (Bayindir and Kavanagh, 2018). Users of SNSs of different functions manifested different behavioral characteristics (Montag et al., 2015; Montag et al., 2018). Facebook is still the largest social network site with respect to user numbers, but it falls behind YouTube in terms of its weekly visiting rate; YouTube leads in the visiting rate by 7% over other SNS, although it has fewer users (Bayindir and Kavanagh, 2018). According to the “2018 Annual Data Report of WeChat,” published by Tencent, 45 billion WeChat messages are sent every day. However, the average sojourn time of a single visit is obviously shorter than that of “Headline,” an SNS for sharing news and content. Most WeChat friends have a realistic basis, and individuals prefer to use WeChat to satisfy their need for society and affection (Gan, 2017, 2018). WeChat Moments is a function that allows users to share their life and posts on the WeChat application. The main users of WeChat are Chinese, yet there are more than 200 million WeChat users from other countries. Although cultural factor of trusting beliefs plays a role in the self-disclosure decisions of users from various cultures (Krasnova et al., 2012), the need for social communication and the motivations for using social media applications are consistent throughout the world. Therefore, the current study could be generalized to a large extent.

A friend may know a person through the information shared on his or her WeChat Moments, so WeChat Moments is also used to promote users’ real social identities. Users of WeChat Moments mainly share mood notes, as well as notes related to learning and working, daily chores, cuisines, travel, etc. The posting patterns that are available to users include text and picture, as well as text and photos or short videos, which may enhance communication effects. Through WeChat Moments, on the one hand, users find emotional support via interpersonal communication with friends; on the other hand, they shape their own images through shared information. As a smartphone-based social networking site, its rich and diverse features further highlight the fragmentation in information sharing and acquisition. At the same time, WeChat Moments also helps friends overcome the limits of time and space in socialization.

Before social networking sites had gained widespread popularity, studies on computer-mediated communication (CMC) suggested that anonymity in cyberspace, with an absence of reality, could promote expression (Joinson, 2001, 2007; Butler et al., 2009). However, an anonymous identity may have different impacts on users’ disclosures behaviors (Chen et al., 2016). Information sharing in various online contexts has been used as a way to enhance users’ real-world relationships (Subrahmanyam et al., 2008). With its deep integration of networks and real societies, the cyberspace of real societies is equipped with a network communication space based on realistic relationships, and it plays an increasingly important and extensive role in providing a convenient communication platform for people’s work and life (Ibrahim, 2010). The most representative example of such a cyberspace in China is WeChat.

Communication online always comes with more or less different levels of anonymity (Joinson, 2007) Anonymity has been considered in studies concerning free speech online (Akdeniz, 2002). Researchers have gradually put emphasis on the information to which users are exposed as a consequence of users posting about their own lives (Utz, 2015). However, there have not been many studies focusing on how anonymity influences user behavior (Hite et al., 2014), especially in the SNS context. Unlike previous studies (Jessup et al., 1990; Joinson, 2001; Reinig and Mejias, 2004; Lapidot-Lefler and Barak, 2012; Yoon and Rolland, 2012; Hsieh and Luarn, 2014), the current study focuses on the application scenario of WeChat and, in particular, answers the following research questions concerning private SNS:

RQ1. How does perceived anonymity influence users’ intrinsic motivations and information-sharing behaviors on private SNS?
RQ2. Do intrinsic motivations mediate the relationship between perceptions and information sharing on private SNS?

Based on the self-determination theory, we synthesized the existing literature to build a theoretical model, which posits that familiarity exerts negative effects on anonymity perception and positive effects on the intrinsic motivation of information sharing, while perceived anonymity exerts negative effects, and intrinsic motivations mediates the effects among familiarity, perceived anonymity, and information sharing. The current study makes two principal contributions to the literature: first, it scrutinized the negative effect of perceived anonymity on information sharing – an effect that is totally mediated by intrinsic motivation on private SNS. Second, a theoretical model was constructed with which to study both the impact of intrinsic motivation on information sharing and the influence of the basic psychological needs of familiarity and perceived anonymity.

This article consists of the following sections: section “Theoretical Background” reviews the theoretical foundation, section “Research Model and Hypotheses” proposes the research model and hypotheses, section “Methodology” explains the methodology, section “Data Analysis and Results” presents the results and related analysis, section “Discussion” discusses the influences of this article on future research and practice, limitations and potential avenues for future research, and section “Conclusion” concludes the article.

## Theoretical Background

### Anonymity in Computer-Mediated Communication

Computer-mediated communication^1^, has two significant features: anonymity and social context cue deficiency (Hsieh and Luarn, 2014). The communication on SNS is also a kind of CMC, promoting the accumulation of social relations (Neves, 2013). These SNSs are useful for users, who are opposed to meeting new people, because they allow them to enhance their relationships in the real world and maintain contact with their friends, families and other connections (Ellison et al., 2007). Offline social capital also impacts SNS use (Yoon, 2014). In China, WeChat has been deeply integrated into users’ work, learning, and everyday lives (Lien et al., 2017; Gan, 2018). However, users still face the challenge of balancing interpersonal relationships and potential privacy concerns (Lowry et al., 2011; Hallam and Zanella, 2017). Verifying the applicability of relevant theories will require an additional examination of how anonymity influences SNS behavior.

Anonymity is when a person’s real identity is unknown due to a lack of identity and information identification in the social interaction (Marx, 1999). Anonymity online means that one cannot find out another’s identity or one cannot be identified by others (Christopherson, 2007), which provides a suitable approach to the study of SNS, because this concept is closely related to human relations research on communication on the Internet (Scott and Orlikowski, 2014). Identity anonymity and visual anonymity have impacts on human behavior on SNS (Lapidot-Lefler and Barak, 2012; Rainie et al., 2013). People may be more willing to share content and messages with an anonymous online identity, without worries about the consequences of their actions (Joinson, 2007; Levmore and Nussbaum, 2010; Jardine, 2015).

The emerging online communication has resulted in intense debates on the pros and cons of anonymity. There are substantial studies that discuss the positive or negative effects of anonymity on SNS (Christopherson, 2007; Lapidot-Lefler and Barak, 2012; Scott and Orlikowski, 2014; Fox et al., 2015; Jardine, 2015; Chen et al., 2016, 2019). Some studies have suggested that anonymity on SNS is a significant way to protect private information (Brazier et al., 2004; Rainie et al., 2013) and build a personal image (Lin et al., 2017), and it has been suggested that people use anonymous online forums as platforms for self-disclosing actions that they feel guilty about (Levontin and Yom-Tov, 2017). However, in the contemporary Internet environment, with a significant use of smart mobile phones, the influence of anonymity on behavior requires further in-depth exploration, as there is no absolute anonymity in the current online environment (Bodle, 2013). Indeed, the data produced by mobile phone networks can identify most people from only pieces of information from their social media use or from the location from which they make calls (De Montjoye et al., 2013).

The influence of CMC anonymity on behavior has been extensively studied by researchers, which has led to theories that illustrate anonymous behavior (Christopherson, 2007). The social identity model of the deindividuation (SIDE) effect asserts that, in the context of anonymous identities, people show a behavioral tendency to obey a group norm due to the prominence of an individual identity (Vilanova et al., 2017). Anonymity in computer-mediated communication may not necessarily lead to antisocial behavior (Christopherson, 2007). Instead, anonymity could enhance social processes related to group identity in online communication (Spears, 2017). While anonymity may benefit information sharing, there is an argument that it may negatively affect sharing behavior (Yoon and Rolland, 2012). The negative effects of online anonymity are reflected in the effects of personalization, misconduct, and false information, which are related to the dark side of the Internet (Fox and Moreland, 2015). In Internet-based interpersonal communication, such as social networking sites, anonymity can also play a negative role in information exchanges (Yoon and Rolland, 2012; Chen et al., 2016, 2019). Research on Twitter also showed that anonymous participants expressed greater hostile sexism through tweeting than non-anonymous participants (Fox et al., 2015).

The hype personal model emphasizes people’s tendency to strategically construct their self-image using Internet technology, making use of online messages to enhance their relationships (Walther, 2007). SNS provides real-time feedback for the construction process of users’ personal images and enhances the ability of users to build their own image and influence others (Li et al., 2018). The hype person is a more perfect version of oneself than reality, and WeChat users take advantage of the impression management technology and relationship maintenance functions, as a mediated interface that WeChat strategically permits to achieve their goals and needs (Zhang et al., 2017).

### Motivation and Self-Determination Theory

The self-determination theory explains the determination process of behavior, where individuals make decisions only by their own desires, without being influenced by any other external factors (Deci and Ryan, 1985). It is a theory of self-motivation, delving into human motivation and individual personality with conventional empirical methods, which puts emphasis on how psychology impacts people’s personalities and the way they evaluate their own behaviors (Ryan and Deci, 2017). Three basic human psychological needs have been defined (Ryan and Deci, 2017): autonomy, with which one feels self-motivated when behaving; competence, indicating an individual’s need to feel competent, while engaging in activities; and relatedness, one’s desire to maintain relationships with friends, family, etc. Based on the theory of self-determination, social contexts can enhance individuals’ self-motivation by internalizing their external motivations and maintaining good behavior by satisfying their needs of autonomy, competence, and relatedness. These three critical psychological needs are closely related to users’ behavior (Ryan and Deci, 2017).

Some studies have confirmed the influence of intrinsic motivation on different types of information sharing on SNSs. The influence of intrinsic motivations on the sharing of knowledge related to intentions and behaviors in firms has been confirmed (Welschen et al., 2012; Lai and Chen, 2014; Rode, 2016; Safa and Von Solms, 2016), as well as actual travel experience sharing on social media (Kang and Schuett, 2013), m-coupon sharing (Tang et al., 2016), and sharing information about products and services (Cho et al., 2015). The crowding-out effect was found when examining users’ motives to share commercial content on social networking sites: in self-reports, intrinsic motives for sharing dominated, while in the experiment, extrinsic incentives induced a greater willingness to share (Vilnai-Yavetz and Levina, 2018). The specific factors that influence the intrinsic motivation of information-sharing behavior have been refined (Welschen et al., 2012; Lai and Chen, 2014).

While some studies have explored the role of other factors in promoting intrinsic motivation, such as system support and information feedback (de Almeida et al., 2016). There is a gap in the literature relating to the influence of psychological perception factors on intrinsic motivation. Furthermore, Research examines the motivation of sharing behaviors on SNS using the theory of planned behavior (Cho et al., 2015; Shibchurn and Yan, 2015; Chen et al., 2018). However, there is a lack of research on the influence of intrinsic motivation on information-sharing behavior on SNS from the perspective of self-determinism.

Information-sharing behaviors in the WeChat Moments function are forms of self-expression that are determined by individual competence levels (Wang et al., 2016). Social relatedness, based on private social networking and convenient communication, is an important component of WeChat usage (Gan and Wang, 2015; Gan, 2017). WeChat use intensity can significantly predict users’ motivations, and intrinsic use motivation was the mediator between the use intensity and subjective well-being of users (Wen et al., 2016). SNSs are perfect for users to express themselves, since they relate to the gratification of self-efficacy (Cheung et al., 2015; Vogel and Rose, 2016). On SNSs, information sharing is a self-motivated behavior. Thus, we hypothesize that the satisfaction of basic needs will impact users’ information-sharing behavior on WeChat.

## Research Model and Hypotheses

**FIGURE 1:**
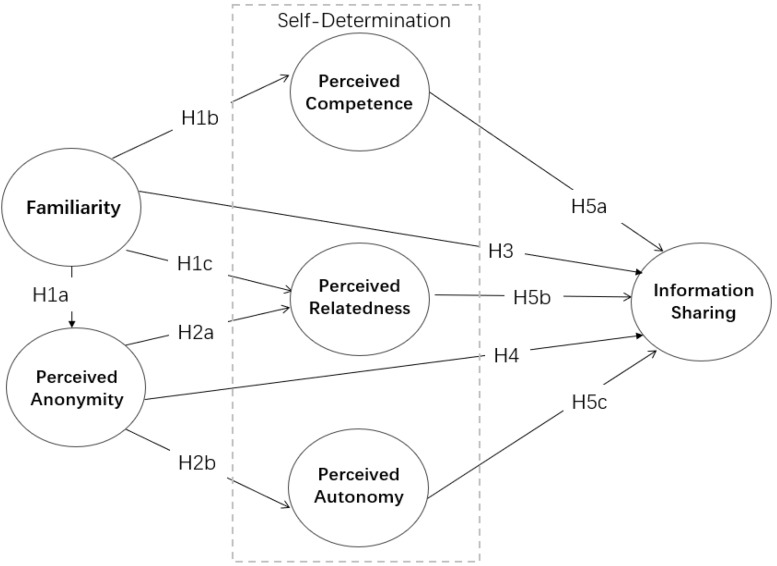
Research model.

The current study constructs a model based on the self-determination theory, which is informed by the most popular SNS, WeChat Moments, in China. We examine how the factors of familiarity and perceived anonymity affect the motivation to share information on WeChat. In addition, this study also pays attention to the mediating influence of self-determination on familiarity, perceived anonymity, and information-sharing behavior. The research model is depicted in Figure 1.

### Familiarity and Anonymity on Social Networking Sites

Familiarity and anonymity are two interrelated but different psychological perceptions that have an influence on users’ behavior. Familiarity refers to an understanding of a thing, contact, or interaction experience that forms a relatively stable cognitive structure and carries the expectation of possible situations and results in subsequent contact with someone or the use of something (Kaptein et al., 2013). Social networking sites provide major platforms for exhibitionism, and users can share information at their own will (Munar, 2010). Besides, they can also adjust the words and pictures of their posts to present themselves (Moon et al., 2016). SNSs allow users to present themselves and connect with friends in different ways. Thus, they serve as venues for significant signals of identity, which enables users to perform properly in the virtual world and articulate friendship links (Ellison et al., 2011). The familiarity of the platform reflects the integration of WeChat contactors with users’ real social relations. Thus, we hypothesize that:

H1a: Users’ familiarity will have a positive impact on their perception of their anonymity on WeChat Moments.

Familiarity is a concept referring to one’s previous associations, connections and knowledge, which is closely related to trust (Komiak and Benbasat, 2006). Familiarity influences how individuals understand others’ behavior and actions on SNSs (Sánchez-Franco and Roldán, 2015). Users’ previous experiences with computers directly enhance their self-efficacy. They believe that computers are user-friendly and useful (John, 2013). Their sense of familiarity with the Internet and expectations of its outcomes has a positive relation with how users perceive their self-efficacy in using SNSs (Eastin and LaRose, 2000; Kenny et al., 2012). Some perceptions related to identifiability, such as the degree of familiarity, perceived similarity with previous experiences, and trust have a positive relation with users’ participation in online communities (Zhao et al., 2012). Thus, we hypothesize that:

H1b. Users’ familiarity will have a positive impact on their perception of their competence on WeChat Moments.

Familiarity may reduce uncertainty, such that people can feel closely related to each other and supported by the people around them (Lin and Utz, 2017). With respect to acquaintances, users have a higher mutual trust and closer ties, and increasing trust is a necessary requirement for online self-disclosure (Taddei and Contena, 2013). WeChat is an acquaintance social platform with a reality-based form as its foundation (Wang and Gu, 2016). Thus, the degree of familiarity therein will affect people’s awareness of their relationships (Finkel et al., 2015). With familiarity, uncertainty will be lowered (Gefen, 2000), enabling people to feel that they are capable of adequately using this platform to make contact with others and feel support from others. Thus, we hypothesize that:

H1c. Users’ familiarity will have a positive impact on their perception of relatedness on WeChat Moments.

Perception of anonymity refers to a low level of identifiability and results in users’ self-perception that they are untraceable in their virtual interactions (Kang et al., 2013). Under anonymous conditions, social bonds are less strong, and social norms are more often aggressive (Wright, 2014). When in group discussion, it was revealed that users who feel anonymous are more likely to embellish others’ opinions, compared with those who are identifiable (Jessup et al., 1990). On social networking sites, anonymous participants reported more post-task sexism than identifiable participants (Fox et al., 2015). The anonymous behavior indicated the consequence that interpersonal connections are weakened in the virtual world. Not being identified means that there is no familiar social connection. Thus, we hypothesize that:

H2a. Users’ perception of anonymity will have a negative impact on their perception of relatedness on WeChat Moments.

Perception of anonymity introduces security awareness into interpersonal interactions (Bodle, 2013). Researchers have posited that the context of communication influences receiver attempts to identify sources, as well as their perceptions of senders (Rains and Scott, 2007). The anonymity of in-group members has been shown to cognitively enhance the perceived unity or entitativity of groups (Sassenberg and Postmes, 2002), which results from reductions in psychological perceptions of individual self-mastery. Users are strategic about using social networking sites to improve their images in social relations (Vogel and Rose, 2016). When users perceive themselves as highly anonymous, they may think about others’ reactions to their sharing, and these thoughts may reduce individuals’ perceived autonomy (Yoon and Rolland, 2012). Thus, we hypothesize that:

H2b. Users’ perception of anonymity will have a negative impact on their perception of autonomy on WeChat Moments.

Familiarity means a close relation, cultural identification, and trust (Gefen, 2000). Concepts related to familiarity, such as interface and habitualness, have been shown to play a critical role in information sharing (Yu et al., 2010; Kim, 2016). The development of an ongoing, trusting relationship, with an understanding of people’s needs, concerns, and shared responsibility, has a positive influence on information-sharing behaviors (Yang and Maxwell, 2011). In addition, familiarity may reduce the perception of risk in communication, mediating the impact of changes in policies’ monetization options on whether people would like to reveal information (Gerlach et al., 2015). The online interrelation built upon personal reputation is crucial for encouraging successful knowledge sharing (Hung et al., 2011). In addition, the role of social capital and a directed social network in users’ information sharing on SNSs has been highlighted (Liu et al., 2016). Thus, we hypothesize that:

H3. Users’ familiarity will have a positive impact on their information-sharing behavior on WeChat Moments.

The concept of perceived anonymity refers to how much personal identity information users believe has been revealed (Hite et al., 2014). Anonymity promotes some negative online behavior (Barlett et al., 2016). Research also suggests that a higher level of perceived anonymity on SNSs means less tendency toward self-disclosure (Chen et al., 2016). There are supportive results proving the negative relationship between anonymity and knowledge-sharing behavior in virtual communities (Yoon and Rolland, 2012), as well as a significant relationship between privacy concerns and self-disclosure on Facebook (Faisal and Alsumait, 2011). On WeChat, users interact and keep in touch with one another to gain attention and a sense of existence and connection by sharing information (Lien et al., 2017). Identifiability is important for users to maintain relationships with their acquaintances, so unidentifiability may reduce the tendency of users to share information. Thus, we hypothesize that:

H4. Users’ perception of anonymity will have a positive impact on their information-sharing behavior on WeChat Moments.

### Self-Determination Factors of Information Sharing on Social Networking Sites

The relationship between intrinsic motivation and behavior in humans is supported by studies on online communities (Yoon and Rolland, 2012; Wang and Hou, 2015). We assume that satisfaction, based on the intrinsic needs of the self, affects users’ information-sharing behavior in maintaining relationships and building their image.

The level of task performance has been shown to be greatly influenced by how competent individuals believe they are. Self-perceived competence has emerged as a significant predictor of willingness to communicate (Donovan and MacIntyre, 2004). Self-efficacy has various positive impacts and has hence become a significant variable in social psychological research (Gecas, 1989). It has emerged as a highly effective predictor of motivation, learning, and performance (Chen et al., 1998) and has also been shown to have an effect on behaviors associated with sharing knowledge on online platforms (Hsu et al., 2007), as well as product information sharing on SNS (Cho et al., 2015; Hajli and Lin, 2016). Thus, we believe that competence has become a driving force behind users’ information sharing on SNSs. Thus, we hypothesize that:

H5a. Users’ perception of their competence will have a positive impact on their information-sharing behavior on WeChat Moments.

Perceived relatedness defines how connected they feel with the people around them (Yoon and Rolland, 2012). SNSs provide fundamental platforms, where people can build connections on the Internet (Lin and Lu, 2011). With the spread of SNSs, users feel closely connected with more people, such that they can accumulate social capital and obtain more information (Boyd and Ellison, 2007; Ellison et al., 2011; Zhao et al., 2012; Yoon, 2014). In addition, the social interaction ties of trust, rules of reciprocity, and identification influence how people share with others on online platforms (Chiu et al., 2006), and the effect of relationship building through SNS on an individual’s intention to share information has been confirmed (Kim, 2012). Thus, we hypothesize that:

H5b. Users’ perception of relatedness will have a positive impact on their information-sharing behavior on WeChat Moments.

Perceived autonomy is a concept that shows how much control a person thinks he/she has over his/her own actions. When individuals are in a virtual world, their level of self-presence and autonomy are two critical factors (Jung, 2011). Users’ can adjust their behaviors strategically, such that they can enhance their relationships. The more autonomy they believe they have in maintaining their friendships, the more satisfied they are (Demir et al., 2011). Sharing behavior may depend on people’s own self-expressive needs (Constant et al., 1994), of which the logical premise is the user’s independent control of the information expressed. A user’s personal status is also significant for the information diffusion on social networking sites (Stieglitz and Dang-Xuan, 2013). Users who were driven by the potential satisfaction of their information seeking were more likely to share on social networking sites (Lee and Ma, 2012). Positive attitudes toward sharing were found to lead to positive intentions and, ultimately, to actual sharing behaviors (Bock and Kim, 2001). SNS users’ structural autonomy has been confirmed as an antecedent of relational inertia in information sharing (Cheon et al., 2015). Thus, we hypothesize that:

H5c. Users’ perception of their autonomy has a positive impact on their information-sharing behavior on WeChat Moments.

### The Mediating Effect of Self-Determination Factors

Human motivation is a complicated, multidimensional internal process (Deci and Ryan, 1985; Chen and Jang, 2010). Several studies have confirmed that self-motivation has direct relations with psychology and behaviors (Charbonneau et al., 2001; Prabhu et al., 2008; Zapata-Phelan et al., 2009; Fernet et al., 2013). According to the theory of self-determination, self-motivation is correlated with three basic needs: whether people feel competent, the degree of relatedness, and whether there is self-autonomy (Deci et al., 1989). While there are many studies investigating these three factors, their mediating roles in an online context, especially on social networking sites, have rarely been empirically tested. In computer and human interactions, perceptions of users’ competence mediate their use behavior (Canary and Spitzberg, 1989; Davis and Wiedenbeck, 2001). In addition, the mediating effect of some self-determination factors has been confirmed in some SNS contexts (Li et al., 2013, 2014; Wong et al., 2015). Thus, we hypothesize that:

H6. Perceived competency and relatedness have a mediating effect on familiarity and information-sharing behavior on WeChat Moments.

H7. Perceived relatedness and perceived autonomy have a mediating effect on anonymity and information-sharing behavior on WeChat Moments.

## Methodology

This study puts emphasis on WeChat Moments users’ information-sharing behaviors. WeChat Moments provides users with virtual spaces to share information with their existing friends, which is an ideal platform for the current research on information sharing.

In addition, WeChat was chosen due to the surge in its popularity since its introduction. WeChat is now the most popular acquaintance-based social networking site, having around 1 billion unique active users, of which more than 200 million users are from countries other than China (Tencent, 2018). WeChat has the typical features of an acquaintance social platform, and more than 70% of Chinese people maintain numerous social relations on WeChat (Wang and Gu, 2016). We collected our data from frequent users, which are believed to constitute an appropriate and representative sample of acquaintance-based social networking sites.

### Data Collection

In the preliminary stages, this study tested the research model through an investigation of WeChat users from China. The data were collected based on an online questionnaire using the online survey platform, www.sojump.com. The purposive sampling method was applied in this survey. Participants were enrolled through a representative sample service, where they were asked to fill in a questionnaire online. After they finished the questionnaire, they received a coupon and had the chance to win something through a lucky draw. Screening questions were included as a filter in order to identify frequent WeChat Moment users for the study, which yielded a total of 531 valid questionnaires out of 601 responses (Supplementary Table S1). Table 1 below shows the results of the survey concerning the participants’ characteristics.

**TABLE 1:** Descriptive statistics of respondent characteristics.

**Measure**	**Value**	**Frequency (%)**
Gender	Female	332 (62.5)
	Male	199 (37.5)
Age (years)	18–25	144 (27.1)
	26–30	150 (28.2)
	31–40	184 (34.7)
	41–50	39 (7.3)
	Older	11 (2.1)
User history	<2 years	14 (2.6)
	2–3 years	65 (12.2)
	3–4 years	147 (27.7)
	4–5 years	128 (24.1)
	>5 years	177 (33.3)
Frequency of using WeChat	>10 times/day	296 (55.7)
	5–10 times/day	219 (41.2)
	1 time/day	9 (1.7)
	<1 time/day	7 (1.3)
Frequency of sharing in WeChat Moments	>2 times/day	72 (13.6)
	1 time/day	74 (13.9)
	>2 times/week	214 (40.3)
	1 time/week	60 (11.3)
	Less	111 (20.9)
Who see your WeChat Moments^∗^	People you know in real world	484 (91.1)
	Friends you are familiar with	459 (86.4)
	Colleagues at work	427 (80.4)
	Online friends have never met in the real world	197 (37.1)
	Nearly none of them will see	2 (0.4)

*^∗^Items are the multiple-choice questions with more than one correct answer, and the rest only has one right answer.*

The distribution of sample demographic information is relatively reasonable. Although bias differences exist in the gender distribution of the research, the factors discussed in this study have some similarity with the researched population. Gender differences do not affect the researches on general sharing behaviors online, which are confirmed by some related studies on similar gender distribution differences (Yoon and Rolland, 2012; Cheung et al., 2015; Yan et al., 2016). The respondents’ WeChat Moments views are characteristic of users of acquaintance SNSs. The respondents are familiar with the use of WeChat and often share information on WeChat Moments.

An ethics approval was not required, according to the relevant institutional and national guidelines and regulations, and the informed consent of the participants was implied in their completion of the survey. First, the data collection of the current study needs no fuzzing processing, as no privacy or sensitive issue is involved. Second, the data collection of the current study does not involve implication, drug or mental manipulation, as the subjects are only required to report their experience and behavior tendency according to the use conditions of the online platform. Thus, no issue with respect to safety, health or right and interest protection is involved. Third, the data extraction of the current study only withholds users’ IP addresses, as there is no need to record private information concerning, for example, the names and identities of the subjects. Fourth, the questionnaire for collecting the data for the current study includes an informed consent statement, and the subjects are only requested to answer questions according to their use conditions. The data are only used for scientific research, and no influence is generated on the privacy, reputation, living conditions or health of the participants. Fifth, the current study does not store or use the private information of the participants, and any information that may lead to identity risks (only the IP Address) is removed during the analysis and submission for scientific review.

### Measures

All applied measures of this study were drawn from the relevant existing literature. We translated the scale into Chinese and then translated it back into English, based on the research needs. We asked two researchers with a Ph.D. to verify the consistency of the expressions in the scale in order to ensure that the translation was consistent with the original scale. Multi-item measures were applied to make sure the study is valid and reliable. All measures used five-point Likert scales (1 = strongly disagree to 5 = strongly agree). Table 2 lists the constructs and measures applied in this paper.

**TABLE 2:** Psychometric properties of measures.

**Items**	**Loading**	***t*-value**	**Mean**	**SD**
***Familiarity*** (*Cronbach’*α = *0.610; CR* = *0.772; AVE* = *0.459) (Gefen, 2000)*				
FA1: I am familiar with searching for people in my WeChat Moments (dropped)				
FA2: I am familiar with how to share content in WeChat Moments	0.685	19.83	3.96	0.88
FA3: I am familiar with reading shared content in WeChat Moments	0.672	19.58	3.83	0.91
FA4: I am familiar with the interaction with familiar people in the WeChat Moments	0.715	23.58	3.89	0.86
FA5: I am familiar with the use of WeChat Moments	0.632	17.04	4.32	0.82
***Perceived anonymity*** (*Cronbach’*α = *0.822; CR* = *0.864; AVE* = *0.560) (Hite et al., 2014)*				
PA1: I believe that people who can see my WeChat Moments do not know who I am	0.661	11.34	2.28	0.89
PA2: I think that people in my WeChat Moments do not know my real identity	0.721	13.90	2.20	1.01
PA3: My WeChat Moments can easily tell people who I am^∗^	0.800	23.46	2.78	1.11
PA4: People in my WeChat know my real identity^∗^	0.736	17.01	2.28	0.92
PA5: My real personal identity can be guessed or know by people who can see my WeChat Moments^∗^	0.789	25.21	2.52	1.05
***Perceived competence*** *(Cronbach’ α = 0.722; CR = 0.829; AVE = 0.550) (Deci et al., 1989)*				
PC1: I am capable of using WeChat and WeChat Moments well	0.613	16.79	4.25	0.74
PC2: Sharing information with WeChat Moments gives me a sense of accomplishment	0.732	28.91	3.40	0.95
PC3: Interacting with others using the WeChat Moments makes me feel that I am a capable person	0.785	36.54	3.81	0.92
PC4: I often feel that I am competent when socializing in the WeChat Moments	0.815	51.03	3.51	0.95
***Perceived relatedness*** *(Cronbach’*α = *0.775; CR* = *0.856; AVE* = *0.597) (Deci et al., 1989)*				
PR1: I really like the people in my WeChat Moments	0.739	31.52	3.72	0.79
PR2: The people in my WeChat Moments care about me.	0.791	42.67	3.55	0.96
PR3: The people in my WeChat Moments are friendly toward me	0.767	37.04	3.68	0.91
PR4: I feel a lot of closeness and intimacy in WeChat Moments	0.789	40.16	3.59	1.00
***Perceived autonomy*** *(Cronbach’*α = *0.698; CR* = *0.815; AVE* = *0.525) (Deci et al., 1989)*				
PO1: I feel that I can express opinions and share contents freely in the WeChat Moments	0.766	32.23	3.85	0.86
PO2: I feel that I am more of myself in the WeChat	0.725	24.84	3.30	1.01
PO3: I can control over what I want to express following my own wishes in the WeChat	0.704	23.91	3.97	0.91
PO4: I can be what I want to be in the WeChat Moments	0.697	23.35	3.59	1.04
***Information sharing*** *(Cronbach’*α = 0.700*; CR* = 0.815*; AVE* = 0.525*) (Lin et al., 2009)*				
IS1: I often express some real opinions, ideas, or feelings in WeChat Moments	0.715	25.70	3.83	0.88
IS2: I often interact with others in the WeChat Moments	0.758	31.86	3.73	1.00
IS3: I share a variety of contents in the WeChat Moments, not necessarily focusing on a subject	0.644	18.32	3.93	0.93
IS4: I often share various contents in my WeChat Moments to people	0.771	34.10	3.50	1.11

*^∗^Reversed scale.*

## Data Analysis and Results

The structural equation model is used to verify the research model. We performed statistical analysis using the partial least squares method (PLS). Smart PLS version 3 (Ringle et al., 2014) has been used to test the research model and is an analytical technique widely used in behavioral and business research because it provides a flexible method, which is widely used by researchers studying human behavior, with coherent explanations for complex relationships (Henseler et al., 2014). PLS is very suitable for predictive exploratory modeling and research (Henseler et al., 2014). According to the two-step analysis method (Hair et al., 2014), we first tested the validity of the measured values and then evaluated the structural model.

With the application of the two-step analytical approach, we were able to confidently draw the conclusion that a structural relationship could be drawn from a series of measurement instruments with desirable psychometric properties.

### Reliability and Validity of the Measurement Items

In order to validate the measurement model, we tested the convergence and discriminant validity of the data. The following general criteria are used to evaluate the convergence effectiveness of the structure: all project loads should be greater than 0.60; the composite reliability (CR) should be at least 0.70; and the average variance extraction (AVE) should be at least 0.50 (Fornell and Larcker, 1981). The results of this study, shown in Table 2, satisfy all three convergence validity conditions, except that the AVE of familiarity is less than, but very close to 0.5, which is acceptable for empirical research. The factor loadings of all items surpassed 0.60, and a single item’s loading lower than 0.60 was excluded.

Discriminative validity refers to the degree to which the measurement of one variable does not reflect other variables. The low correlation between interest measures and other structural measures indicates the validity of the discrimination (Fornell and Larcker, 1981). If the AVE square root of each construct is greater than its correlation with all other constructs, it is proved to be discriminant. As shown in Table 3, the AVE square root of each construct is greater than the correlation of them and all of the other constructs. The results show that all of the measured values have sufficient discriminant validity. In the current study, the evidence proves that our data analysis results have enough higher convergent validity and discriminant validity.

**TABLE 3:** Correlation matrix and psychometric properties of key constructs.

	**FA**	**PA**	**PC**	**PR**	**PO**	**IS**
Familiarity (FA)	(0.678)					
Perceived Anonymity (PA)	–0.178	(0.748)				
Perceived Competence (PC)	0.564	–0.166	(0.742)			
Perceived Relatedness (PR)	0.478	–0.240	0.565	(0.773)		
Perceived Autonomy (PO)	0.464	–0.234	0.564	0.550	(0.724)	
Information sharing (IS)	0.509	–0.220	0.585	0.589	0.548	(0.725)

*The SQRT (AVE) in ( ), Off-diagonal elements are the correlations between constructs.*

### Structural Model

**FIGURE 2:**
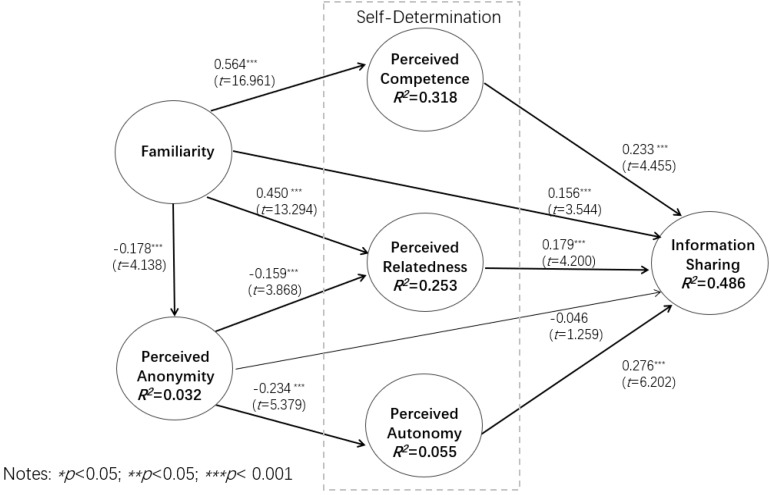
Results of the research model.

Based on the hypothetical research model, we analyze the structural model by examining the significance of the path coefficients and the (*R*^2^) variance for the dependent constructs. Figure 2 reveals the path and its importance for the structural model, as well as the coefficients of each related structure, their *T* values on the structural model and the deterministic coefficients (*R*^2^). From the statistical data, anonymity perception has no direct significant impact on users’ information sharing, while other structures have significant impacts. With the exception of H4, all other hypotheses have been confirmed. We also conducted a half-way data analysis test on the samples, and the results were completely consistent.

The results indicate that the independent variable explains a relatively high proportion of the variance of the dependent variable. In this model, 31.8% of the variance of self-competence perception was explained, and 25.3% of the variance of correlation perception and 48.6% of the variance of information sharing were also explained. The model can moderately predict information-sharing behavior, perceived competence, and perceived relatedness. The predictive effectiveness for perceived autonomy was quite low (*R*^2^ = 0.055), as well as that for perceived anonymity (*R*^2^ = 0.032). The *R*^2^ statistic may be small, yet the coefficient *p*-values can still be statistically significant. Such a relationship between the predictors and response is also very important, even though it may not explain a large amount of variation in the response (Chin, 1998).

According to Hair et al. (2016), the use of PLS-SEM for theory testing and confirmation is limited since it does not have an adequate global goodness-of-fit measure. Researchers using PLS-SEM may not draw on a global goodness-of-fit measure to evaluate the overall model fit, as PLS-SEM focuses on prediction (Hair et al., 2014).

### Mediating Effect Test of Self-Determination

Preacher and Hayes (2004) proposed that the bootstrapping method can effectively and reasonably verify the non-direct impact. In this study, we used this method to verify the mediating effect. As shown in Table 4, the mediating effect of self-determination has been confirmed.

**TABLE 4:** Bootstrapping analysis for the mediating effects of self-determination.

**Effect Types**	**Effect mean**	**SE**	**95% CI**	
			
			**Lower**	**Upper**
FA (total)	0.393	0.040	0.328	0.462
FA → IS (direct)	0.155	0.044	0.084	0.230
FA → IS (total indirect)	0.238	0.032	0.187	0.290
FA → PC → IS	0.132	0.032	0.081	0.186
FA → PR → IS	0.079	0.020	0.048	0.114
FA → PA → PO → SD	0.012	0.005	0.006	0.021
FA → PA → PR → SD	0.005	0.002	0.002	0.009
PA (total)	–0.144	0.040	–0.207	–0.074
PA → IS (direct)	–0.048	0.037	–0.110	0.014
PA → IS (total indirect)	–0.096	0.020	–0.130	–0.065
PA → PR → IS	–0.029	0.010	–0.047	–0.014
PA → PO → IS	–0.067	0.016	–0.095	–0.041

*CI – confidence Interval, 1000 bootstrap samples. SE – standard error.*

Perceived competence and relatedness have crucial and positive mediating influences on familiarity, with respect to the total mediating effect of the perceived competence and perceived relatedness combination. The mediating effect of perceived competence accounted for 33.6% of the total effect, occupying 55.5% of the total intermediary effect. The mediating effect of perceived relatedness accounted for 20.1% of the total effect and 33.2% of the total intermediary effect. The combined effect of perceived anonymity, perceived competence, perceived autonomy, and perceived relatedness comprised 60.6% of the total effect. Familiarity accounted for about 40% of the direct effect on information sharing.

The negative mediating effects of perceived autonomy and perceived relatedness on perceived anonymity and information sharing were significant. This was also evidenced by the total mediating effect on perceived anonymity and information sharing. The intermediary effect of perceived autonomy accounted for 69.8% of the total intermediary effect. The mediating effect of perceived relatedness accounted for 30.2% of the total intermediary effect. In the case when the direct effect of perceived anonymity and information sharing was weakened, perceived autonomy and perceived relatedness had a complete mediating role. As a result, the negative effect of perceived anonymity on information sharing – an effect that is totally mediated by intrinsic motivation – has been confirmed.

## Discussion

The results of current study showed that familiarity has a deep positive influence on perceptions of competence and relatedness and information sharing. The current study also revealed the negative effect of perceived anonymity on users’ motivations in information sharing on WeChat Moments. It showed that the higher the level of anonymity perceived by the users, the less relatedness and autonomy they feel. Motivation had a partly mediating impact on familiarity and information sharing and a total mediating impact on anonymity and information sharing. We also found that self-motivation, perceived competence, perceived relatedness, and perceived autonomy significantly affect information-sharing behavior.

The direct connections between perceived anonymity and information-sharing behaviors on acquaintance SNSs are not significant. This might be explained by the fact that the effect of perceived anonymity is intermediated by perceived relatedness and perceived autonomy. Many scholars have studied the correlations in how anonymity affects behavior in many different contexts (Hite et al., 2014). Indeed, CMC research has confirmed that anonymity influences behavior (Reinig and Mejias, 2004). Anonymous online settings are also related to a tendency to provide socially desirable responses (Muhlenfeld, 2005). However, the influence of different types of anonymities, especially perceived anonymity, on behavior still needs to be examined in different online contexts. The current study showed that the negative effect of perceived anonymity on information sharing is totally mediated by intrinsic motivation.

### Theoretical Implications

The current study presents implications to facilitate future explanation of the behavior of the users of social networking sites, particularly WeChat Moments. Whetten (1989) argues that when existing models are applied to a new context, they lose their academic significance, unless they are modified. An important way to promote the development of a theory is to study relevant problems from the perspective of other fields, so as to further explain the mechanism of the relationship between different variables. The current study makes theoretical contributions in the following ways.

First, these findings supplement previous studies on the information-sharing behavior on WeChat Moments. The results showed that familiarity has an important positive effect on information sharing on WeChat Moments. The findings of the study have important implications for understanding social networking sites based on the acquaintance relationship.

Second, this study further examined the online anonymous behavioral theory. Anonymity has generated obvious differences in the influential mechanism of self-expression behavior between people in acquaintance relationships on WeChat and in other relationships in cyberspace, such as public forums (Yoon and Rolland, 2012; Gan and Wang, 2015). To some extent, the environment of WeChat Moments highlights users’ real social identities and leads to the sharing of positive content (Chen et al., 2016), while perceived anonymity reduces users’ willingness to share information. From the perspective of technological applications, interactions based on social networking sites strengthen the effect of hyper personal communication. A close connection between the subjects of sharing behavior helps to cultivate the construction of hyper-personal impressions. The current study lays the foundation for the further verification and extension of the hyper-personal communication theory in relation to WeChat Moments.

Third, the effectiveness of the self-determination theory in explaining WeChat Moments was validated. In addition, the influence of the basic psychological requirements of familiarity and anonymity was validated. The partial intermediary effect of intrinsic motivation on familiarity and information sharing, as well as the total intermediary effect of intrinsic motivation on anonymity and information sharing, was confirmed. These variables could be applied to studies on the self-determination theory in other contexts. The research model is applicable to more online scenarios and is capable of considering other psychological or cultural factors.

### Practical Implications

The findings of this study also have some practical utility, especially for policymakers, service providers, and users of social networking sites. It is shown that familiarity has an important effect on perceived competence, perceived relatedness, and information sharing on WeChat Moments. For service providers, SNSs should put more emphasis on the design of the human–computer interaction interface to meet users’ preferences, facilitate the investment in and bonding of user relationships, and increase the means of user interactions to promote feelings of familiarity. They should also encourage users to invite other people with which users have real-world social relationships in order to introduce more acquaintances into social networking sites. The improvement of user activation and connection will further increase the value of WeChat Moments for individual users.

The current study suggests that perceived anonymity has a negative influence on perceived relatedness and autonomy, and the two intrinsic motives totally mediate the negative influence of perceived anonymity on information sharing. Service providers should include more information concerning user identity on WeChat Moments to decrease perceived anonymity. In this way, they could improve the degree of individual identity, enhance self-awareness and generate the use of acquaintances to advance public self-awareness; both qualities are connected with low deindividuation and aggression (Prenticedunn and Rogers, 1982). How to improve the supervision of net users’ real identity should not be the only focus of regulators. It is also critical to consider the influence of real identity on perceived anonymity and include it in the user identity regulation system. That perceived anonymity has different effects on user behavior in different online spaces should be taken into consideration.

### Limitations and Future Directions

This study has the following limitations. First, the research scenario and participants of the survey have been restricted to WeChat Moments. As information sharing is considered a dynamic process and is affected by individual and contextual factors (Hsu et al., 2007), additional research is needed to examine how and to what extent contextual differences affect users’ information-sharing behavior. The research model is applicable to more online scenarios and is capable of considering other psychological or cultural factors. Second, the current study provides no support for the H5 hypothesis, which suggests that perceived anonymity has no direct effect on information sharing, and the two intrinsic motives totally mediate the negative influence of perceived anonymity. Familiarity, a positive psychological perception, directly affects information sharing, while anonymity, a negative psychological perception, can only affect information sharing through motivation. We have repeatedly verified this phenomenon, although its causes need further verification. Third, differences exist in the age and sex distribution of the research sample and the whole netizen group. Future studies should enhance the representativeness and universality of the research sample and consider more variables in future study. Finally, this study did not differentiate the impacts of different types of information. Individuals have different preferences for different information types, which may influence their sharing and acquisition behavior. Future studies should therefore consider the characteristics of information, as well as different types of social networking sites.

## Conclusion

The current study applied the theory of self-determination and constructed a research model to explore the influence of two psychological factors, familiarity and perceived anonymity. We looked into how these two factors impact the self-motivation of users in information sharing on WeChat Moments. It has been proved that this measurement model has high validity in terms of both its convergent and discriminant aspects. The basic elements of intrinsic motivation displayed a critical intermediary effect. With the prevalence of smartphones, mobile Internet-based social networking services have become an important medium for communication. Social networking sites for acquaintances are playing an increasingly crucial role in social reality. The current study contributes to the literature by allowing for an understanding of familiarity, anonymity, intrinsic motivation, and information sharing in relation to WeChat Moments. It is hoped that the current study will inspire future researchers to explore human behavior, as it is manifested on WeChat Moments.

## Data Availability Statement

All datasets generated for this study are included in the article/Supplementary Material.

## Author Contributions

XC performed the theory analysis and design, and contributed to drafting the manuscript. MS analyzed the data and improved the empirical analysis. DW analyzed the data and improved the writing. XS improved the writing and conclusions.

## Conflict of Interest

The authors declare that the research was conducted in the absence of any commercial or financial relationships that could be construed as a potential conflict of interest.

## Footnotes

1 Computer-mediated communication refers to communication using devices such as computers and smartphones as media. Most online communications are considered CMC.

## Supplementary Material

TABLE S1 Questionnaire results.
